# Trailblazers in foregut surgery: Kyle Perry, MD (Ohio State University) interviews John Hunter, MD (Oregon Health and Science University)

**DOI:** 10.1007/s00464-025-11824-z

**Published:** 2025-05-29

**Authors:** Kyle Perry, John Hunter

**Affiliations:** 1 Ohio State University, Columbus, USA; 2 Oregon Health and Science University, Portland, USA

After nearly 30 years of laparoscopic anti-reflux surgery, there are so many fellows and residents that have been trained to perform these procedures. Over the course of these years, fellows and residents continue to provide ongoing training to others in anti-reflux procedures. What is clear from SAGES Masters sessions in Foregut Surgery at the annual meetings, is there are certain technical aspects of the procedure that are passed along through the ongoing Minimally Invasive Surgical training. SAGES has identified several Trailblazers in Foregut Surgery, and the following are transcripts of conversations with the founding experts in anti-reflux surgery. These were video discussions that occurred in 2019, and SAGES would like to memorialize this series as they offer truly valuable experiences.

All readers will find these interviews interesting, from experts to trainees. We may be able to identify through these transcripts whose style of foregut surgery we follow. The Trailblazers were interviewed by members of the SAGES Foregut Committee during the 2019 SAGES Annual Meeting.

**Kyle Perry: I’m Kyle Perry, and I am an Associate Professor of Surgery at Ohio State University. I’m here today to interview Dr. John Hunter for SAGES’ Trailblazers in Foregut Surgery series. Dr. Hunter is a Professor of Surgery at Oregon Health and Science University and the CEO of the OHSU health system. He has also served as a mentor to many surgeons, including me. It’s my pleasure to be here today to talk with Dr. Hunter.**

John Hunter: Kyle, it’s indeed an honor.

**The first thing that I want to ask is this: early in your training and career, did you always see yourself becoming a foregut surgeon, or did that evolve over time? Also, who were the mentors who influenced your career?**

No, I trained as a GI surgeon. My mentors during my residency at the University of Utah were John Dixon, who was really a founder of SAGES and one of the first to see the endoscope as the way forward in surgery. My other mentor was Frank Moody, who was a prominent GI surgeon with interest in the pancreas and biliary system.

I started my career as a biliary surgeon. My interest in endoscopy led me to do many upper GI endoscopies, which resulted in detecting a lot of reflux disease without a good way to treat it. At that point in history, we were only allowed to use PPIs for a maximum of 6 weeks at a time. So, when patients came off PPIs, they would be incredibly symptomatic and say, “What else can you offer me, doc?” This experience got me and my colleagues interested in esophageal disease.

**Early on, as laparoscopic techniques were coming into use in foregut surgery and elsewhere, did you view that as a natural progression of existing open surgeries or as a major paradigm shift that was entirely changing the way surgery was being performed?**

Some of the rules of open surgery also applied in laparoscopic procedures, such as the concepts of the operation, the structures that you could not injure, and the goals of therapy. But because you were entirely dependent on visual optics without tactile sense or use of your fingers in laparoscopy, every operation had to be modified.

**As laparoscopic procedures were emerging, did you envision minimally invasive surgery evolving into what it has become today, or did you see it differently at that time?**

That’s a great question. I saw that laparoscopy would be of use for straightforward operations, such as laparoscopic appendectomy, hernia repair, cholecystectomy, fundoplication, and possibly repair of large hiatal hernias. We did those very early in the development of laparoscopy. I don’t think that I would ever have anticipated a laparoscopic Whipple, esophagectomy, or some of the other major procedures that are done laparoscopically today.

**As that evolution has occurred, is there anything that seems most surprising to you or that you didn’t envision in the evolution of flexible endoscopy?**

I think the use of a flexible endoscope was how we started. Following my residency at the University of Utah, my training involved going to Massachusetts General Hospital to do a flexible endoscopy fellowship. We always thought that the natural orifices provided the best access because no incisions were needed. And so, endoscopy seemed appropriate to the extent that we could do more invasive procedures with a flexible endoscope, such as a POEM (per oral endoscopic myotomy), for example, which was very much in line with doing the PEG (percutaneous endoscopic gastrostomy), an endoscopic procedure from several decades earlier. So, that was very consistent with the way I saw things developing.

The thing that most surprised me was the development of robotics and its penetration of the market. I had a firm sense that although the robotic platform was very intuitive and easy to learn for surgeons who didn’t know laparoscopy, robotics wouldn’t demonstrate any evidence-based benefit that would allow it to thrive. I thought that the cost would be prohibitive given the lack of evidence of benefit.

In many respects, had prostatectomy not come along, robotic surgery might not be where it is today. Clearly, it's easier and better to do a robotic prostatectomy. But the evidence of benefit still isn’t there for many of the other applications for which robotics is being used, and it’s hard to predict what will happen or where it’s going.

My concern and my plea are that surgeons should still learn laparoscopic techniques, because those techniques are translatable to almost any environment. You don’t need a robot to do them. They’re less expensive, and they’re faster. Some training is required to do laparoscopic operations, but so also is some training required to learn how to play the piano—but one needs to do that.

**As we’ve been discussing, the major evolution has been in the complexity and the way in which we apply some of these minimally invasive techniques in laparoscopy. If you look back at the treatment of foregut diseases, are there any things that we previously did as standard of care that now seem particularly cringeworthy or bothersome?**

I think that the evolution of laparoscopic surgery of the esophagus has been very straightforward. The simplest operation, perhaps, was the fundoplication, which then moved on to the myotomy, which moved on to the diverticulectomy, which moved on to smooth muscle tumor resection. Then this progressed to esophagectomy through transhiatal, 2-field, and ultimately 3-field techniques. That’s all very straightforward. I don’t know that there is anything cringeworthy. I do have concerns about the durability of some of the endoscopic procedures. We have written about some of those concerns regarding endoscopic antireflux procedures and have been principal investigators on studies that have demonstrated their benefits, but we don’t know about the longevity of those benefits.

I do have some concerns about placing a foreign body around the esophagus and what will happen over the course of 3, 4, or 5 decades after the procedure. We know that some of them are coming out, and some of them still seem to work. I don’t think it’s cringeworthy, but that is something that I worry about.

The last thing I’d say is that a fundoplication done well— either a partial or total fundoplication—provides excellent reflex control, has great durability (with a failure rate of about 1% per year), and can be redone if it fails. So, I’m not sure what we’re trying to fix there.

**Do you think that maybe the biggest Achilles’ heel in a fundoplication is the durability issue (perhaps along with the side effects, which I think is a debatable point), even with the failure rate being what it is? I think about how superior fundoplications are when they’re done properly.**

**We have a fairly high rate of people going back on PPIs after fundoplication, particularly young people who are having this operation. Are there things that have been done or can be done to improve the durability of the operation?**

I think the most important thing in someone who is on a PPI is to ask whether they are responsive to the PPI. If they’re not responding to the PPI—and we have shown in the *New England Journal of Medicine* article that came out last week that roughly 40% of patients are not responsive to PPIs—then we should study them and find out how many are refluxing.

Again, in the study that we published last week in the *New England Journal of Medicine*, only 20% of these patients are refluxing. So, the first thing I would like to do is to take 80% of those patients off PPIs. Of the 20% who are refluxing, I would then assess whether there is a structural impairment in the Nissen fundoplication. If there is a structural impairment, I’d recommend a redo operation. If there is no structural impairment, which is rare, then you’ve got a bit of a head-scratcher.

**The last thing that I wanted to ask about is what you see as the next frontier in minimally invasive surgery, given the continual evolution and change that you’ve seen in this field during your career.**

That’s a good question. The thing that I have advocated to SAGES for several years (and SAGES has done a great job with this) is to make sure that the institutions that train residents have fellows who have been well trained to become new faculty members who will train these residents to do laparoscopic surgery.

I really enjoyed watching the transition of laparoscopic skills expanding from being specific to a very focused group of specialists, such as you and me, to being more broadly available. The emergency general surgeons—largely trauma surgeons—are now very capable with laparoscopic appendectomy, laparoscopic cholecystectomy, etc. They’re probably the best at getting stones out of the bile duct. They’re very proficient at doing laparoscopic common bile duct exploration, because that’s what they do.

I think that encourages generalizing laparoscopic training so that the skill level increases to the point where most surgeons can do most things laparoscopically. That’s our goal. I don’t see laparoscopy going away. I don’t see it being replaced by robotics, although I know that some individuals would like that to happen. I certainly think that if there is a less expensive robotic platform, it will be very interesting to see what that does. But I don’t think that laparoscopy is a transition state to something better yet.

**I thought of 1 more thing that I want to ask if you will indulge me.**

Yeah.

**If you could give 1 piece of advice to medical students or junior residents who are embarking on a career in this field, what would that be?**

Practice, practice, practice. I learned my skills by teaching courses in laparoscopic surgery. At the end of the course, we would go and work on the stomach and esophagus, doing anastomoses, doing suturing activities, working on suturing skills.

It is like playing the piano—you don’t get better at playing the piano by doing it occasionally. You do it in a concerted fashion every day for a period of time until you’re very good at it. So, use the simulators, use the box, use whatever you have to do, and just continue to practice whenever you have free time between cases or at night when you’re on call and things aren’t going on.

**Alright. Thank you so much for taking the time to do this. It’s been an absolute pleasure.**

It was a pleasure for me. Thank you very much, Kyle.

